# Clinical practice guidelines in Brazil – developing a national programme

**DOI:** 10.1186/s12961-020-00582-0

**Published:** 2020-06-17

**Authors:** Verônica Colpani, Sérgio Candido Kowalski, Airton Tetelbom Stein, Anna Maria Buehler, Daniel Zanetti, Gabriel Côrtes, Edison Vieira de Melo Junior, Jorgiany Emerick Ebeidalla, Natiela Beatriz de Oliveira, Renata Leborato Guerra, Sarah Nascimento Silva, Bruce B. Duncan, Maicon Falavigna, Holger Jens Schünemann

**Affiliations:** 1 Research Projects Office, Hospital Moinhos de Vento (HMV), Rua Ramiro Barcelos, 910, 3° andar, Porto Alegre, RS 90035-001 Brazil; 2 Department of Internal Medicine, Division of Rheumatology, Universidade Federal do Paraná (UFPR), Curitiba, PR Brazil; 3 Graduate Program in Health Sciences, Universidade Federal de Ciências da Saúde de Porto Alegre (UFCSPA); Program in Health Technology Assessment, Grupo Hospitalar Conceição, Porto Alegre, RS Brazil; 4 Health Technology Assessment Unit, Hospital Alemão Oswaldo Cruz, São Paulo, SP Brazil; 5 Department of Management and Incorporation of Health Technology (DGITIS), Executive Secretariat, National Committee for Health Technology Incorporation (CONITEC); Secretariat of Science, Technology and Strategic Inputs (SCTIE); Brazilian Ministry of Health, Brasília, DF Brazil; 6 CONITEC; Plenary member, Special Secretary of Indigenous Health (SESAI), Brazilian Ministry of Health, Brasília, DF Brazil; 7 DGITIS; Executive Secretariat, CONITEC; Secretariat of Science, SCTIE, Brazilian Ministry of Health, Brasília, DF Brazil; 8 General Coordination, Environmental Health Surveillance (CGVAM); Department of Environmental Health, Worker and Emergency Public Health Surveillance (DSASTE); Secretariat of Health Surveillance (SVS); Brazilian Ministry of Health, Brasília, DF Brazil; 9 Health Technology Assessment Unit (NATS), National Institute of Cancer (INCA), Rio de Janeiro, RJ Brazil; 10 School of Medicine, Hospital de Clínicas de Porto Alegre (HCPA), Instituto de Avaliação de Tecnologia em Saúde (IATS), Universidade Federal do Rio Grande do Sul (UFRGS), Porto Alegre, RS Brazil; 11 IATS, Universidade Federal do Rio Grande do Sul (UFRGS), Porto Alegre, RS Brazil; 12 Department of Health Research Methods, Evidence and Impact and of Medicine, McMaster GRADE Centre, McMaster University Health Sciences Centre, Hamilton, Canada

**Keywords:** guideline development, evidence-based medicine, Brazil, clinical practice

## Abstract

In Brazil, governmental and non-governmental organisations develop practice guidelines (PGs) in order to optimise patient care. Although important improvements have been made over the past years, many of these documents still lack transparency and methodological rigour. In order to conduct a critical analysis and define future steps in PG development in Brazil, we carried out a structured assessment of strengths, weaknesses, opportunities and threats (SWOT analysis) for the development of a national guideline programme. Participants consisted of academia, methodologists, medical societies and healthcare system representatives. In summary, the PG development process has improved in Brazil and current investments in methodological research and capacity-building are ongoing. Despite the centralised processes for public PGs, standardised procedures for their development are not well established and human resources are insufficient in number and capacity to develop the amount of trustworthy documents needed. Brazil’s capacity could be strengthened and initial efforts have been made such as the adoption of standards proposed by world-renowned institutions in PG development and enhancement of the involvement of key stakeholders. Further steps involve the alignment between health technology assessment and PG processes for synergy and the development of a national network to promote the interaction between groups involved in the development of PGs. The lessons learned from this paper could be used to foster debate on guideline development, especially for countries facing similar threats on this topic.

## Introduction

The public healthcare system for 209 million Brazilians is based on the principles of universal access, comprehensive coverage, equity, decentralisation and social participation [1]. In Brazil, 100% of the population have access to healthcare through the Brazilian Unified Public Healthcare System (Sistema Único de Saúde; SUS) and approximately one-quarter of those have additional private health insurance coverage or pay directly for services received [2].

The Brazilian Ministry of Health (MoH) develops national practice guidelines (PGs) for health professionals and policy-makers based on technologies that are used across the SUS in order to establish standards for the diagnosis and treatment in public healthcare settings. New technologies are first subject to a systematic assessment by the National Committee for Health Technology Incorporation (CONITEC) [3, 4]. This committee is composed of stakeholders from different health-related sectors of society (including members of the MoH, members of health regulatory agencies and representatives of national health councils). The CONITEC advises the MoH on policies regarding the incorporation and removal of technologies, which include decisions for the coverage of medicines, diagnostic tests, products and procedures in the SUS. This process is supported by health technology assessment (HTA), including systematic reviews of the effects of technologies, cost-effectiveness analysis and budget impact assessment. If the CONITEC’s decision results in the coverage of a new technology by the public health system, CONITEC requests the update of Brazilian MoH guidelines in order to allows its implementation [3–5]. Thus, PGs play a crucial role in Brazilian healthcare delivery. Moreover, they require integration with HTA, which presents a unique opportunity for collaboration between key stakeholders in the health system. This may be used as a model for other countries wishing to both improve care and save resources by increasing cost-effectiveness through evidence assessment and implementation processes.

### The current guideline development process in Brazil

In Brazil, governmental and non-governmental organisations, such as professional societies and the MoH, develop PGs. While PGs developed by the former primarily aim to inform healthcare professionals about best practices, MoH PGs also aim to standardise practices for the public health system. Different departments in the MoH may produce documents with healthcare recommendations, but PGs developed by CONITEC have a normative role, defining the available technologies and circumstances for their implementation in the SUS. These PGs are developed by academic and healthcare institutions, commissioned by the MoH, responsible for evidence review and guideline panels. The PGs are reviewed by CONITEC, ensuring that recommendations are aligned with services currently provided by the public health system, and CONITEC may request modifications or even a new HTA assessment if a new technology is recommended.

However, in Brazil, many PGs lack transparency and methodological rigour [6–9] and do not strictly follow the standards recommended by the Institute of Medicine and the Guidelines International Network (GIN) [10–13]. Moreover, PGs developed by health professional societies often receive funding from the pharmaceutical industry, creating potential conflicts of interest. Nevertheless, the country is experiencing an increasing need for trustworthy recommendations and is trying to improve the PG development process by enhancing the required methodological rigour and transparency [14].

The Brazilian MoH develops PGs through its own technical staff from different Secretariats as well as hiring institutions, such as federal universities and hospital-based HTA units, to develop them. It is responsible for the approval of the delivered products after public consultation. This public consultation includes making a preliminary version of the guideline available online to gather input from society.

**Fig. 1:**
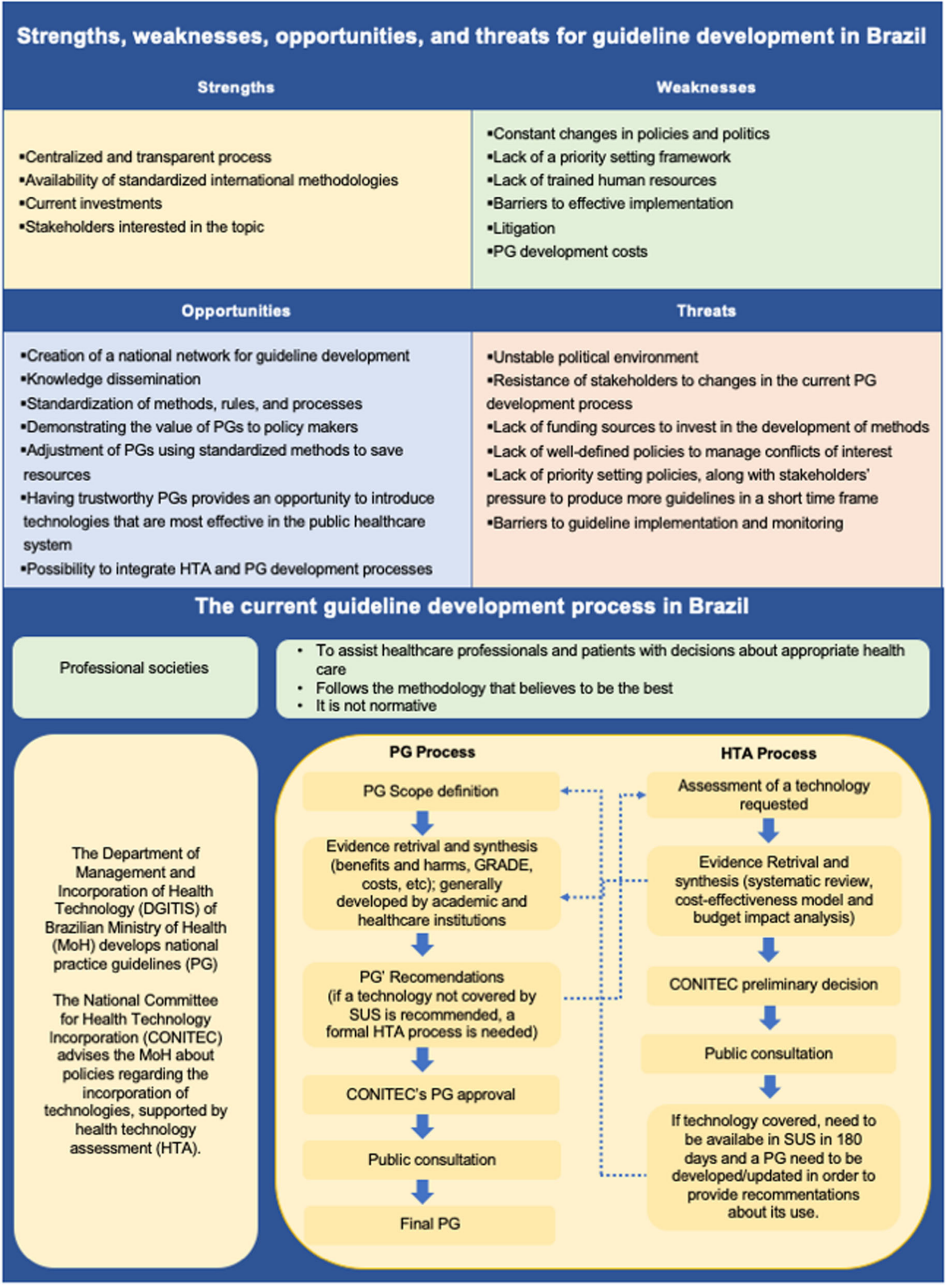
Key aspects of the current scenario and future directions to enhance PG development in Brazil. *PG* practice guideline, *HTA* health technology assessment

Given the importance and potential influence of PGs on healthcare delivery to the 150 million Brazilians who receive care exclusively through the public healthcare sector, there is a need to keep up with best practices in guideline development and implementation and to define the next steps for Brazil. Concerned with the growth and improvement of PG development methodology and in an attempt to bring together professional societies and the public healthcare sector, the authors of this paper conducted a critical analysis and defined the necessary future actions by key players in Brazil. We used a systematic approach (Fig. 1), the basis of which is presented in Additional file 1.

### Strengths, weaknesses, opportunities and threats for guideline development in Brazil

An important strength of PG development in Brazil is that its healthcare system uses a centralised process for guideline development and approval, which leads directly to the implementation of PG recommendations in the public sector. The Department of Management and Incorporation of Health Technologies (DGITIS) of the Brazilian MoH is responsible for managing and coordinating activities related to the development of guidelines for the public healthcare system. This process is followed by a CONITEC assessment and public consultation, and the document is finally approved by the responsible manager of the SUS and officially published. As these PGs are official documents with guidance for healthcare professionals and policy-makers, they shorten the gap between the development and implementation of recommendations in the public health system. Additionally, the representative composition of the CONITEC increases the legitimacy and transparency of the recommendations, as does the public consultation process.

The Department of Management and Incorporation of Health Technologies and the CONITEC were only created in 2011 and, although core activities are well established and implemented, especially those related to HTA, some of their processes are still in the early stages of development and refinement. As in any newly formed organisation that is linked to the government, constant changes in policies and politics can negatively affect the flow of activities. The process can be further jeopardised if there is strong external pressure from stakeholders, such as pharmaceutical industry, medical societies and patient organisations, especially in the absence of well-defined policies governing conflicts of interest. Thus, the development of a sound conflict of interest policy may minimise inequities, save resources, and reduce the likelihood of errors and biases in the process. For example, some stakeholders, as medical societies, may adopt an independent reviewer board for the approval process and require disclosure of conflicts of interest prior to the panel meeting as well as for publication along with the guideline to maximise editorial independence. In addition, as there is a need for long-term policies in the field of guidelines, the presence of uncertainties of the continuity of investments in PG development programmes is a major potential pitfall on achieving the desired outcome, which includes investments in methodological research and capacity-building.

Despite the centralised processes, the procedures for PG development are not well established. Although there are several guiding documents and experts available in the country, the methods used in the development of PGs in Brazil need to be improved. The professionals involved in the process should be following international standards, such as those of GIN or the Institute of Medicine, and use established approaches to assessing evidence and develop trustworthy recommendations. Additionally, more research should be carried out to evaluate the quality of PGs in order to assess its improvement over time [10, 11, 15]. For example, developing guidelines is a complex process that requires standardised methods and specific knowledge of epidemiology, public health, health economics, evidence-based medicine, and group leadership and processes. Currently, human resources are insufficient in number and capacity to efficiently produce trustworthy guidelines on a larger scale. Because the PGs developed by the Brazilian MoH define the technologies that will be covered by the public healthcare system, the lack of updated guidelines results in an increase in litigation as a means of achieving care. In the absence of updated recommendations, patients may obtain the right to an intended intervention through the legal system, a process which circumvents formal evidence-based analysis, resulting in higher costs and suboptimal use of resources. Currently, the cost of litigation accounts for about US$ 3–4 billion in expenses annually, corresponding to almost 10% of the total MoH budget. Additionally, in Brazil, national evidence is lacking for questions related to several neglected diseases, patient and social values, and preferences, costs, equity and acceptability by patients and policy-makers.

Some initial efforts have been made to improve guideline development and translation into health policies in Brazil. The MoH has begun to standardise PG methodology, following standards recommended by world-renowned institutions such as WHO. Continuing to integrate other well-established methods such as GRADE will be key in the improvement of the Brazilian PG process [10–12, 15–17].

In addition, governmental agencies have begun to invest in training and methods development projects, involving key stakeholders (e.g. national organisations, academia, professional societies, healthcare providers, policy-makers, patients and society), in order to improve PG and HTA development. Additionally, the MoH now promotes synergy between HTA and PG development. Although PGs and HTA share important similarities (such as evidence synthesis, a structured decision framework and internal approval processes), they have been developed independently of one another in Brazil. Despite these signals of progress, there is still a long way to go to ensure that the different stakeholders will work efficiently together, particularly with respect to priority-setting and sharing a common agenda for guideline development and method standardisation. Achieving this common agenda will avoid duplication of efforts.

A major current weakness that should be prioritised for action is the language barrier. Documents in Brazil are often published and disseminated in Portuguese, which hinders international collaboration between guideline development teams as well as the worldwide dissemination and critique of Brazilian PGs. However, Brazil must be able to recognise and explore opportunities for partnerships with countries and international organisations that apply proper PG development processes, including guideline adaptation and external support, in order to reduce costs and increase work efficiency. Collaborative regional initiatives may be an option. For instance, Brazil is a member of the Health Technology Assessment Network of the Americas and The International Network of Agencies for HTA, both of which generate opportunities for collaboration and sharing of relevant knowledge with other organisations, especially from low- and middle-income countries. Many low- and middle-income countries face similar political and economic challenges. Collaborations that have been established in the field of HTA may be expanded to guideline development, thus being one of the aims of the GIN International Network of Agencies for HTA working group.

Considering PGs as an evidence synthesis and evidence-based support tool, Brazil is only one of many countries making efforts to change their development process. Improvements have been made, such as the implementation of a centralised process, the use of a standard methodology as GRADE and the investment in training [18–21], but there still are further steps to be taken. Given that one of our goals is to improve PG development and dissemination, it is important as a first step to prioritise topics that focus on the major needs of the population. In the long term, this may reduce inequalities in the treatment of prevalent diseases. Another obstacle is that the guideline development process still depends largely on a few groups of experts in guideline methodology. A lack of funding to stimulate the interest and growth of competence in this field has limited its spread to universities and research institutions, posing a threat to the long-term sustainability of PG development programmes. Furthermore, it is important that, after standardising the methods for PG development and dissemination, strategies for the effective implementation of PGs are required. There are several options requiring a multifaceted approach to the development of user-friendly material by all stakeholders and strategies for the application of PG recommendations to clinical practice.

## Conclusion

The PG development process has improved in Brazil but the country still needs to adopt standard methods and approaches to improve the trustworthiness of its PGs. This analysis, summarised in Fig. 1, presents key aspects of the current scenario and future directions to enhance PG development in Brazil.

Fostering debate on PG development across the country is important to act in synergy with international agencies and to develop better national guidelines following similar initiatives taking place in other advancing countries such as China and India [22–24]. In this context, international collaboration will help in capitalising on the international experience to improve the PG process. This, in turn, should promote the rational use of resources in order to provide the best available technologies for the Brazilian population.

The authors of this article agreed on the main steps that need to be taken to improve PGs in Brazil, including the establishment of a national network for PG development and the standardisation of methods using well-established international methodologies. A collaborative network is needed to avoid the duplication of efforts and align methods and quality standards. This network should enhance the relationship between groups and institutions engaged in PG and HTA development, both at national and international level. This approach of incorporating different groups in the process should promote the development of an integrative environment and nurture a culture that stimulates the sharing of strategies for PG development and use in Brazil.

## Supplementary information

**Additional file 1.** Methods and approach for assessing the status of guideline development in Brazil and defining future directions at the national level.

## Data Availability

Data sharing is not applicable to this article as no datasets were generated or analysed during the current study.

## Abbreviations

CONITEC: *National Committee for Health Technology Incorporation*
HTA: Health Technology assessment
MoH: The Brazilian Ministry of Health
PGs: Practice guidelines
SUS: Brazilian Unified Public Healthcare System
