# Supplementary material for: Clinical practice guidelines in Brazil – developing a national programme
Source: Health Res Policy Syst. 2020 Jun 17;18:69. doi: 10.1186/s12961-020-00582-0 (PMC7302389; doi:10.1186/s12961-020-00582-0)
Supplement: Supplementary file 1 — Additional file 1. Methods and approach for assessing the status of guideline development in Brazil and defining future directions at the national level. [file 12961_2020_582_MOESM1_ESM.docx]

**Additional file 1**. Methods and approach for assessing the status of guideline development in Brazil and defining future directions at a national level.

We convened leading scientists in evidence-based medicine and practice guideline (PG) development, policy makers, scholars, and stakeholders gathered to present ideas on how to improve Brazilian guidelines in terms of methodology and population health impact.

The group was composed by eighteen professionals involved in PG development. Of those, eight were Ministry of Health representatives (DZ, GC, EVJM, JEA, NBO, NMI, PGF, RLG, SNS), one was a medical society representative (SK), three were representatives from Brazilian federal universities (ATS, BBD, JMB) and five were researchers involved in PG development for Ministry of Health (VC, AMB, MF, TG, RLG). The workshop was chaired by an expert in guideline methodology and development of national guideline programs (HJS). In a day-long meeting, we carried out a structured assessment allowing all parties to share their views on the process of guideline development in Brazil. The meeting was held in Porto Alegre, southern Brazil, on 16^th^ of November of 2017, during the seminar “Health Evidence 2017 – The information value: from the science to the decision making”, promoted and financed by the Brazilian Ministry of Health, through the Department of Management and Incorporation of Health Technologies in a partnership with the Institute for Education and Research of Hospital Moinhos de Vento.

The discussions included an analysis of strengths, weaknesses, opportunities, and threats (SWOT) in the development of a national guideline program in Brazil. SWOT analysis is a qualitative method to identify and assess the strengths and weaknesses within a program, as well as the threats and opportunities outside the given program^1^.

Although literature was not formally reviewed for this meeting, the chair and some of the participants are involved in comprehensive guideline methods research, such as Guidelines 2.0 checklist (HJS, SK, MF), G-I-N and WHO guidance (HJS) and five are members of GRADE working group (VC, SK, ATS, MF, HJS)^2-5^.

We present an overview of the process in the Figure below. First, a brief overview of Brazilian and international PG development programs was provided, followed by the presentation of meeting objectives and SWOT methodology. The discussion for each SWOT component was performed independently, with the process consisting of four small group sessions, followed by a large group discussion for group consensus. Small groups were composed by 2 to 3 members with different backgrounds, with member rotation. The process duration for each one of the four components was from 45 to 60 minutes.

**Figure**. Overview of the workshop process.


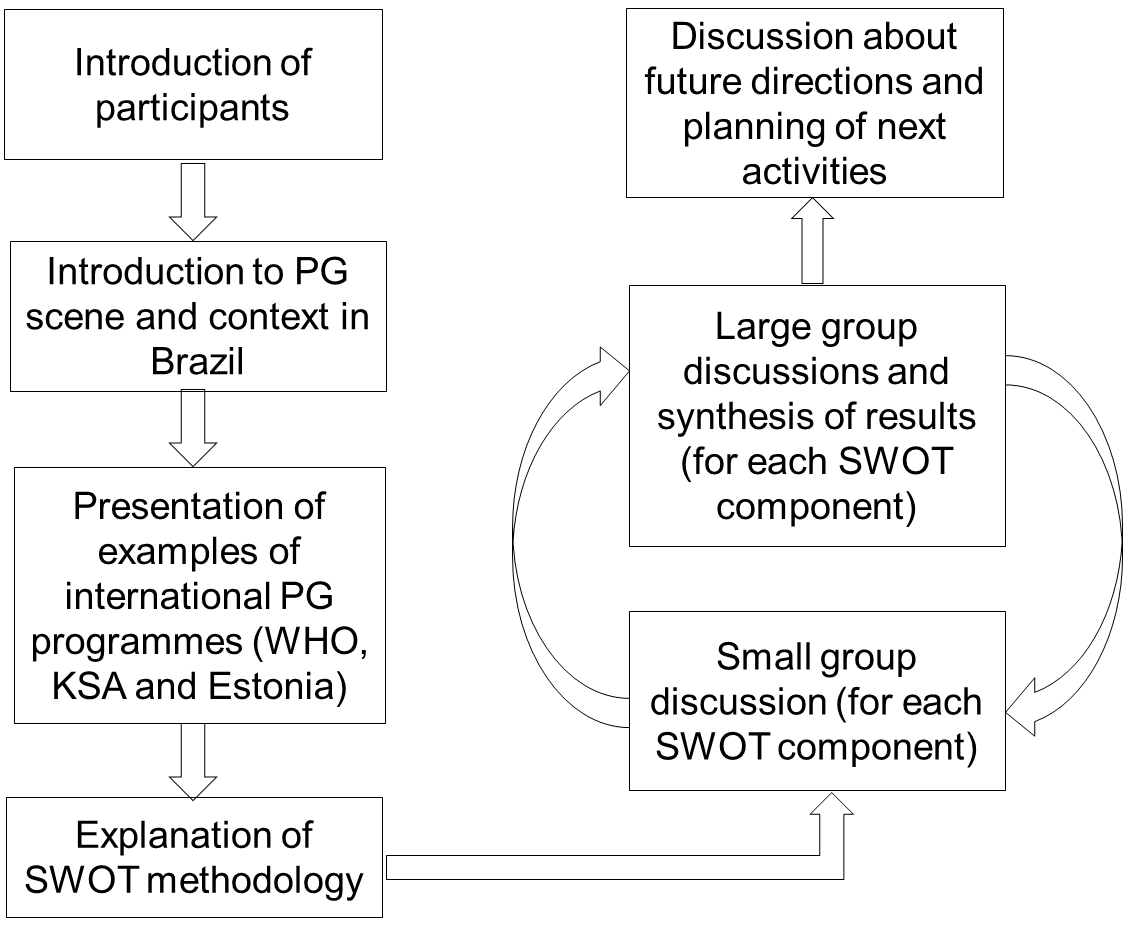


PG: practice guidelines; KSA: Kingdom of Saudi Arabi; SWOT: analysis of strengths, weaknesses, opportunities, and threats; WHO: World Health Organization

**References**

1. van Wijngaarden JD, Scholten GR, van Wijk KP. Strategic analysis for health care organizations: the suitability of the SWOT-analysis. *Int J Health Plann Manage* 2012;27:34-49. doi: 10.1002/hpm.1032

2. Morgan RL, Florez I, Falavigna M, et al. Development of rapid guidelines: 3. GIN-McMaster Guideline Development Checklist extension for rapid recommendations. *Health Res Policy Syst* 2018;16:63. doi: 10.1186/s12961-018-0330-0

3. Schunemann HJ, Fretheim A, Oxman AD. Improving the use of research evidence in guideline development: 1. Guidelines for guidelines. *Health Res Policy Syst* 2006;4:13.

4. Schunemann HJ, Al-Ansary LA, Forland F, et al. Guidelines international network: principles for disclosure of interests and management of conflicts in guidelines. *Ann Intern Med* 2015;163:548-53. doi: 10.7326/M14-1885

5. Schunemann HJ, Wiercioch W, Etxeandia I, et al. Guidelines 2.0: systematic development of a comprehensive checklist for a successful guideline enterprise. *CMAJ* 2014;186:E123-42. doi: 10.1503/cmaj.131237
